# Evaluation of phage—antibiotic combinations in the treatment of extended-spectrum β-lactamase-producing *Salmonella* enteritidis strain PT1

**DOI:** 10.1016/j.heliyon.2023.e13077

**Published:** 2023-01-19

**Authors:** Mona G. Alharbi, Rashad R. Al-Hindi, Ibrahim A. Alotibi, Sheren A. Azhari, Reem M. Farsi, Addisu D. Teklemariam

**Affiliations:** a Department of Biological Sciences, King Abdulaziz University, Jeddah, 21589, Saudi Arabia; b Health Information Technology Department, Applied College, King Abdulaziz University, Jeddah, 21589, Saudi Arabia

**Keywords:** Extended-spectrum beta-lactamase, Phage antibiotic synergism, Siphoviridae phage, *Salmonella* enteritidis, Wastewater

## Abstract

Foodborne infections caused by *Salmonella* spp. are among the most common foodborne diseases in the world. We isolated a lytic phage against extended-spectrum beta-lactam producing *S. Enteritidis* strain PT1 derived from chicken carcass. Results from electronmicrography indicated that phiPT1 belonged to the family, *Siphoviridae*, in the order, *Caudovirales*. Phage phiPT1 was stable at temperatures from 4 °C to 60 °C and inactivated at 90 °C. phiPT1 retained a high titer from pH 4 to pH 10 for at least 1 h. Nevertheless, it displayed a significant decrease (*p* < 0.05) in titer at pH 11 and 12, with phage titers of 5.5 and 2.4 log_10_ PFU/mL, respectively. The latent time and burst size of phiPT1 were estimated to be 30 min and 252 PFU/infected cell, respectively. The virulence of phage phiPT1 was evaluated against *S.* Enteritidis strain PT1 at different MOIs. phiPT1 reduced *Salmonella* proliferation relative to the negative control (MOI 0) at all MOIs (*P* < 0.05). However, there is no significant difference among the MOIs (*P* > 0.05). The phage-antibiotic combination analysis (PAS) indicated that synergism was not detected at higher phiPT1 titer (10^12^ PFU/mL) with all tested antibiotics at all subinhibitory concentrations. However, synergistic activities were recorded at 0.25 × MIC of four tested antibiotics: cefixime, gentamicin, ciprofloxacin, and aztreonam in combination with phage at 10^4^, 10^6^ and 10^8^ PFU/mL (ΣFIC ≤0.5). Synergism was detected for all antibiotics (0.1 × MIC) except meropenem and colistin in combination with phiPT1 at 10^4^, 10^6^ and 10^8^ PFU/mL (ΣFIC ≤0.5). Synergism also displayed at the lowest concentrations of all antibiotics (0.01 MIC) in combination with phiPT1 at all titers except 10^12^ PFU/mL. Such characteristic features make phiPT1 to be a potential candidate for therapeutic uses.

## 1 Introduction

Foodborne infections caused by *Salmonella* spp. are among the most common foodborne diseases in the world [1]. Recently, about 2600 *Salmonella* serovars have been identified [2]. Different serovars of *Salmonella* have different host spectrum and virulence factors [3]. In recent years, although *Salmonella* Typhimurium (*S*. Typhimurium) and *Salmonella* Enteritidis (*S*. Enteritidis) are the most prevalent, other serotypes have emerged. For instance, Lin et al. identified 156 *Salmonella* isolates from chicken carcasses in Taiwan, and among them, *S.* Tennessee (5.1%), *S.* Kentucky (12.8%), *S.* Schwarzengrund (20.5%), and *S.* Albany (41.7%), are the most commonly isolated serovars [4]. Since 1989, *Salmonella* strains which developed resistance to multiple antibiotics especially for first line antibiotics have emerged [5]. This resistance has been a dramatic increase and nowadays shifted towards extended-spectrum cephalosporins and fluoroquinolones [6]. A worldwide increase in cephalosporin resistance has been reported among *Salmonella* spp., particularly *Salmonella enterica* Typhimurium. Several plasmid-mediated β-lactamases are responsible for broad-spectrum cephalosporin resistance, particularly CTX-M-type extended-spectrum β-lactamases (ESBLs) [[7], [8], [9]].

The majority of human infections caused by *S*. Enteritidis occur during food preparation or through consumption of undercooked or raw food, including poultry, chicken, beef, milk, vegetables, and fruits [10]. Due to the rapid increase in antibiotic resistance occurred among *Salmonella* serovars currently alternative strategies such as phage therapy re-emerged and evaluated in *in vivo* and *in vitro* experiments targeting different *Salmonella* serovars mainly those which cause foodborne infections in humans [11,12]. Bacteriophages (phages) are predators of bacteria that are harmless to animals and humans. Several investigations on phage indicated that, phages have shown antibacterial activity against multidrug resistance (MDR) bacterial foodborne pathogens with high specificity and a strong killing effect [[13], [14], [15], [16]]. Salmonella phage Felix O1 was the first to be identified, in the 1930s [17]. Several studies have demonstrated the effectiveness of *Salmonella* phages against different *Salmonella* serovars since that time [[18], [19], [20], [21]]. Researchers have speculated that phages isolated from one country are unlikely to be able to lyse bacteria in another because of their defense mechanisms and high diversity [22] therefore, there is a need for new phages that target specific serovars so that practical applications can be realized.

Several research findings indicated that different types of *Salmonella* bacteriophages have shown promising results against various *Salmonella* strains [23,24]. According to Abdelsattar et al. [25], a lytic phage, ZCSE6, reduced *Salmonella* growth (MOI 1) by 1000-fold in milk following 3 h of incubation at 37 °C. In a different study, two lytic phages, SPHG1 and SPHG3, and their cocktail resulted in a significant reduction in the viable count of *S.* Typhimurium in milk, water, and on chicken breast (at MOIs of 100 or 1000). Based on the results, the cocktail of these two phages could be a potential candidate in biocontrol and/or phage therapy against foodborne Salmonellosis [26]. Similarly, Huang and colleagues reported a truly lytic phages, LPSE1, against *Salmonella* Enteritidis which displayed significant bacteriolytic activity towards the tested strain in ready to eat foods including milk, sausage, and lettuce [27]. Currently, some phage products are available commercially in the market (e.g., Salmonelex™, SalmoFREE**®** and SalmoFresh™) for treating foods that are at high risk for *Salmonella* contamination [28].

To date, researchers studied the combined effect of phage with antibiotics targeting different antibiotic resistant bacterial strains [29,30]. Some of these studies showed synergetic effects while there are reports which indicated additivism, and antagonism relationships [31]. Phage can lower the working minimum inhibitory concentration (MIC) for bacterial strains which already resistant to antibiotics relationships [31]. Phage-antibiotic combination reducing the occurrence of antibiotic as well as phage resistance by reducing the required concentration of antibiotics that is needed in case of individual antibiotic treatment. Moreover, the antibiofilm effects of phage enhanced the therapeutic efficacy of antibiotics when they were delivered simultaneously [32].

Based on our literature search, very limited studies have addressed the synergistic relationship between phage and antibiotics targeting the human pathogenic *Salmonella* strain derived from chicken carcass. Therefore, the aim of this study was to evaluate the combined effect of the newly isolated phage against ESBL producing *Salmonella enterica* subsp. *enterica* serovar Enteritidis strain PT1 (*Salmonella* Enteritidis (*S.* Enteritidis) with some selected conventional antibiotics (gentamicin, ciprofloxacin, ceftazidime (30 μg) and ceftazidime/clavulanic acid, cefotaxime (30 μg) and cefotaxime/clavulanic acid, aztreonam (30 μg) and aztreonam/clavulanic acid, cefepime, cefixime, meropenem, imipenem, ertapenem, piperacillin and tazobactam, amoxicillin–clavulanic and colistin).

## 2 Material and methods

### 2.1 Host bacteria

ESBL producing *Salmonella enterica* subsp. *enterica* serovar Enteritidis (*S.* Enteritidis) strain PT1 which is the cause of foodborne human gastroenteritis was used as host to isolate a lytic phage from a sample collected from Jeddah Wastewater Treatment Plant. This bacterial isolate derived from chicken carcass sold at retail market. The molecular confirmation was made using 16s rRNA genome sequencing. The isolate preserved in 50% glycerol (v/v) at −80 °C and revived in Nutrient Broth (NB) medium at 37 °C overnight.

### 2.2 Antibiotics and sensitivity test

Different classes of antibiotics (Table 1) were selected for antibiotic sensitivity test and PAS analysis. These antibiotics are commonly used to treat human Salmonellosis [33] (Table 1). The antimicrobial profile of the host isolate was determined by disk diffusion assay following the protocols of the Clinical and Laboratory Standards Institute (CLSI) [34].

**Table 1:** Antibiotic disks used for sensitivity test and their respective concentrations [33].

Antibiotics	Concentration μg/disk	Susceptible	Intermediate	Resistant
**Gentamicin**	10 μg	≥15	13–14	≤12
**Ciprofloxacin**	5 μg	≥31	21–30	≤20
**Ceftazidime (30 μg) and ceftazidime/clavulanic acid (30/10 μg)**	30/10 μg	≥18	14–17	≤13
**Cefotaxime (30 μg) and cefotaxime/clavulanic acid (30/10 μg)**	30/10 μg	≥20	15–19	≤14
**Aztreonam (30 μg) and Aztreonam/Clavulanic acid (30/10 μg)**	30/10 μg	≥18	14–17	≤13
**Cefepime**	30 μg	≥25	19–24	≤18
**Cefixime**	5 μg	≥19	16–18	≤15
**Meropenem**	10 μg	≥23	20–22	≤19
**Imipenem**	10 μg	≥23	20–22	≤19
**Ertapenem**	10 μg	≥22	19–21	≤18
**Piperacillin and Tazobactam**	100/10 μg	≥21	15–20	≤14
**Amoxicillin–clavulanic**	20/10 μg	≥18	14–17	≤13
**Colistin**	10 μg	≥74	–	≤4

### 2.3 Wastewater sample collection

A total of 1 L of raw wastewater sample was collected from Jeddah Wastewater Treatment Plant. The sample was transported to King Abdulaziz University microbiology laboratory in an ice box, then stored in a refrigerator until it was processed within 24 h.

### 2.4 Enrichment and isolation of bacteriophages

The raw wastewater samples were spun down at 10,000 ×*g* for 10 min and the supernatant was passed through 0.22 μm proto filters (Fischer Scientific, Ottawa, ON) to remove solid and cellular materials. The filtrate was then directly used for phage isolation [35]. Shortly, 1000 μL of overnight host culture were inoculated into 20 mL of the phage filtrate mixed with equal volume of 2× NB supplemented with 2 mM CaCl_2_ for enrichment. The culture was placed in the shaking incubator at 37 °C, 100 rpm, for 48 h. Afterward, the suspension was spun down at 8, 000×*g* for 12 min at 4 °C and the supernatant was filtered using 0.22 μm size proto filters to exclude non phage products and the filtrates was stored at 4 °C until used.

### 2.5 Purification

A double agar overlay method (DAL) was used to purify phages, as described by Gencay et al. [36]. The positive phage lysates for spot tests were used and one single plaque was picked from the soft layer by touching the single plaque using a paster pipette and placed in 500 mL phosphate buffer saline (PBS = pH 7.5). Afterward, the preparation was kept at 4 °C for proper diffusion of phages into the suspension. Then, the titer of isolated phage determined by DAL. This process is conducted several times until we get morphologically uniform plaques. Lastly, the purified phage filtrates were stored at 4 °C for further use.

### 2.6 Concentration of phages

The full-plate lysate method was used to concentrate phages titer, as others have stated [37,38]. Briefly, the phage lysate containing the clear and uniform plaques was selected and 8 mL of PBS was poured over the top agar. The plate was then kept at room temperature for 12 h with gentle shaking. After that, the phage suspension was aspirated with a 10 mL sterile syringe and passed through 0.22 μm proto filters to remove bacterial debris. Lastly, the final titer of phiPT1 was determined by DAL.

### 2.7 Determination of phage titer and the morphology of plaques

A 10-fold dilution of the purified phage lysate was performed in PBS, and a DAL technique was employed to determine the titer. Plaques were counted manually. To determine the titer, a plate containing plaque counts between 30 and 300 plaques was used. The titer was calculated, and the result recorded as PFU mL−^1^ [39]. Additionally, the plaque's size was measured with a ruler, and photographs were taken with a digital camera.

### 2.8 Efficiency of bacteriophage adsorption

The host bacteria were cultivated in NB at 37 °C overnight. Around 6 mL of the host culture (OD_600_ = 0.3) were spun down and pellets were rinsed with PBS (pH 7.5). The resulting pellet was suspended in 2 mL of NB medium and incubated at 37 °C for 15 min after centrifugation. Then, the purified phage lysate was added at 10 MOI. Samples were collected at indicated times during incubation and centrifuged for 1 min at room temperature (6000 ×*g*). Once the supernatant was obtained, the DAL technique was used to determine the titer of phage in the supernatant. Phage lysates that were added directly to host strain at zero time were considered 100% non-adsorbed phages. Phage adsorption rate were then computed as described by Ref. [38].

### 2.9 Lysis profile assay

The purified phage lysates were individually added at 10 MOI to the host bacterium (OD_600_ = 0.5) at 37 °C. The mixtures were then incubated with shaking at 37 °C, and the density was monitored at the same time intervals (2 h) for 30 h by OD_600_ measurements [40].

### 2.10 Thermal stability assay

The thermal resistance of phiPT1was determined at 40, 50, 60, 70, 80, 90, and 100 °C in a thermal-controlled water bath and at 4 °C in a standard refrigerator. Equal volumes of phiPT1 (10^8^ PFU/mL) and PBS (7.5 pH) were incubated for 1 h as a control. Then, the titer of heat treated phiPT1 were determined by DAL technique [38].

### 2.11 pH stability assay

The pH stability of phiPT1 was assayed in NB with the pH range of 2.0–14.0. This assay was performed at 4 °C for 1 h. Thereafter, the phiPT1 titer was determined by the DAL technique [38].

### 2.12 Host range determination

The lytic range of phiPT1 was investigated as the protocol described by Yang et al. Briefly, overnight cultures of bacterial cells were mixed with molten soft agar (0.7%) and then poured on solid nutrient agar plate to establish double-layered plates. Thereafter, 10 μL of purified phage lysate (>10^8^ PFU/mL) was spotted on the surface of bacterial lawn and incubated overnight for 12 h. The culture dishes were visualized for the presence of lysed zone and positive results further confirmed by DAL [41].

### 2.13 One-step growth analysis

We studied the lytic activities of the isolated phages in a one-step replication cycle experiment following the previously stated protocol with some modifications [42]. Briefly, the host bacterium was grown in NB at 37 °C with shaking until OD_600_ = 0.2. Afterward, 10 mL of culture sample was spun down (4000 ×*g*) for 10 min at 4 °C. Following that, the pellet was resuspended in fresh NB medium. After 5 min of incubation at 37 °C, phiPT1 was added at a MOI of 10. Following a 10 min incubation at 37 °C, non-adsorbed virions were removed by washing 3 times with 1000 μL of NB medium containing 3 mM sodium azide (4 °C, 4000 ×*g*, 10 min). Next, the suspension was mixed with 25 mL of NB (zero time) and incubated in a shaker incubator at 37 °C. The aliquots (100 μL) were computed at 10 min intervals over a 60 min period. The titer of phiPT1 was determined by DAL and then the burst size and latent period were determined.

### 2.14 Phage—antibiotic combination (PAS) assay

#### 2.14.1 Multiplicity of infection (MIC) determination

Microbroth dilution assay was employed to set the MIC for seven antibiotics selected for phage -antibiotic synergistic analysis [43]. In a 96-well microtiter plate, 100 μL of Muller Hinton Broth (MHB) was added and antibiotics were diluted from 0.125 to 128 μg/mL. Equal volume of MHB without antibiotics was used as negative control. The *S.* Enteritidis strain PT1 inocula of 5 × 10^5^ dilutions from the overnight grown cells were added and incubated for 17 h at 37 °C. Then, MIC values were recorded and analyzed using CLSI guideline [44]. The antibiotics breakpoints and concentrations are presented in Table 5.

#### 2.14.2 Quality control

To evaluate the inhibitory effect of the selected antibiotics on phiPT1, 10^12^ PFU/mL of phiPT1 was mixed with 100 μg/mL of each antibiotic and then incubated at 37 °C for 3 h. The bacterial culture without antibiotics served as negative controls. DAL was conducted to determine the change in plaque morphology and phage titer as well [45].

#### 2.14.3 Phage—antibiotic synergy (PAS)

The PAS assay was conducted to determine the synergistic interaction between phiPT1 and the selected antibiotics at three different subinhibitory concentrations, i.e., 0.25, 0.1 and 0.01 of the MIC concentration. In a 96-well microtiter plate, 0.1 ml of MHB containing individual antibiotic was added. phiPT1 were diluted from 10^12^ to 10^4^ PFU/mL 5 μL adjusted *S.* Enteritidis strain PT1 culture (5 × 10^5^ CFU/mL) was added in individual well. Culture dishes were incubated for 17 h at 37 °C, thereafter the cultures were serially diluted (10^4^, 10^6^, 10^8^ and 10^12^) and plated on tryptone soy agar plates, and CFU/mL determined. MHB broth, antibiotic and phage alone considered as control [45].

#### 2.14.4 Fractional inhibitory concentration

Fractional inhibitory concentration (FIC) indices were measured in accordance with the data from the phage antibiotic synergy experiment and with this the combined effect of phage and antibiotics was computed [46] using the following formula:

ΣFIC = FIC_Ab_ + FIC_p_ = (C_Ab_/MIC_Ab_) + (C_p_/MIC_p_)

With MIC, minimum inhibitory concentration; P, phage; C, concentration; Ab, antibiotic; FIC, fractional inhibitory concentration. FIC was interpreted as antagonistic (ΣFIC >2.0), additive (≥1.0 ΣFIC ≤2.0), indifferent (>0.5 ΣFIC ≤1.0), or synergistic (ΣFIC ≤0.5).

### 2.15 Statistical analysis

Data are expressed as means and standard deviation (SD). For the statistical analysis, GraphPad Prism 8.0.1 (USA) was used. A minimum of three replications were performed whenever needed. PAS was analyzed using analysis of variance (ANOVA). *P* < 0.05 was considered statistically significant.

## 3 Results and discussion

### 3.1 Host strain confirmation and antibiotic resistance profile

The partial 16S rRNA based genomic sequence revealed that the host *Salmonella* strain isolated from chicken carcass was 100% identical to *S.* Enteritidis strain PT1 (Accession number - CP043433). Antibiotic resistance pattern of *S.* Enteritidis strain PT1 is shown in Table 2. The isolate developed resistance against 69.2% of the antibiotics tested. The isolate showed resistance for beta-lactam antibiotics which is due to the enzyme, β-lactamase, produced by the pathogen [47]. It is also resistant against fourth and third generation cephalosporin (cefepime and cefixime) but sensitive for carbapenems (meropenem, imipenem, and ertapenem). Observing such a high antibiotic resistance in this study is in harmony with the global emergence of MDR among WHO priority pathogens [48]. This high MDR may be attributed to the frequent and/or miss use of antimicrobials in treating infections and to boost the productivity and growth of farm animals [49]. Horizontal gene transfer contributed a crucial role in the spread of AMR in bacterial communities [50]. The increase in the occurrence of ESBL producing *Salmonella* strains complicate the disease prevention and control approach which ultimately result in death in the society [51]. Such phenomena are the driving force of the present research for using phages as a therapeutic or biocontrol agents for control and prevention of infections and spread of MDR strains.

**Table 2:** Antibiotic resistance profile of *S.* Enteritidis strain PT1.

Category	Antibiotics (μg/disk)	Susceptibility^a^
**Aminoglycoside**	Gentamicin (10 μg)	R
**Quinolone**	Ciprofloxacin (5 μg)	R
**ESBLs production indicators**	Ceftazidime (30 μg) and ceftazidime/clavulanic acid (30/10 μg)	R
	Cefotaxime (30 μg) and cefotaxime/clavulanic acid (30/10 μg)	R
	Aztreonam (30 μg) and Aztreonam/Clavulanic acid (30/10 μg)	R
**Fourth generation cephalosporin**	Cefepime (30 μg)	R
**Third-generation cephalosporin**	Cefixime (50 μg)	R
**Carbapenems**	Meropenem (10 μg)	S
	Imipenem (10 μg)	S
	Ertapenem (10 μg)	S
**Penicillin**	Piperacillin and Tazobactam (100/10 μg)	R
**β-Lactam/β-lactamase inhibitor combinations**	Amoxicillin–clavulanic (20/10 μg)	R
**Polymyxin**	Colistin (10 μg)	S
Total percent resistance	**9 (69.2%)**	

a **,** R: resistant; S: susceptible.

### 3.2 Plaque and virion morphology

**Fig. 1:**
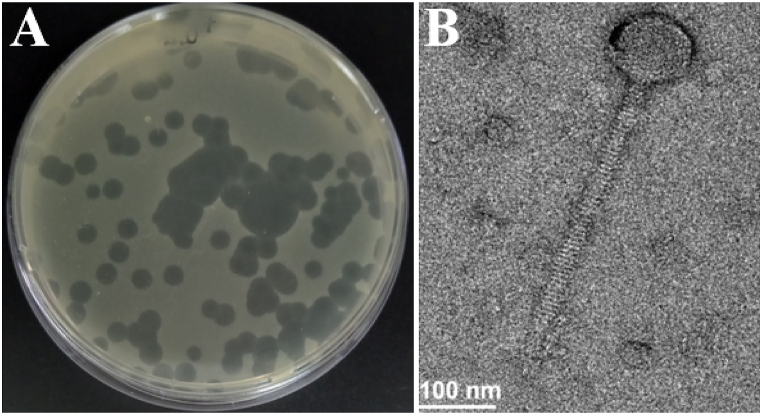
Phage phiPT1 (A) plaques on the lawn of *S.* Enteritidis strain PT1, (B) TEM micrographs of phiPT1.

Several wastewater samples from Jeddah Wastewater Treatment Plant were collected and tested for the presence of phages against *S.* Enteritidis strain PT1 isolated from chicken carcass. In this study, a lytic phage designated as phiPT1 was isolated using the DAL assay and phenotypically characterized using different techniques. According to the results, SEP52 produced clear, round, medium size plaques (1–2 mm) on the lawn of their host (Fig. 1A). This results are in harmony to those reported by Refs. [52,53].

The morphology of virions was studied by TEM and results indicated that phiPT1 displayed an icosahedral head and a long non-contractile tail. The magnitude of head diameter, head and tail length of phiPT1 were 55, 70, and 132 nm, respectively (Fig. 1B). Based to the International Committee on Taxonomy of Viruses, phiPT1 showed typical features of phages belonging to the *Siphoviridae* family in the order *Caudovirales* [54]. According to scientific literature, above 95% of the phages were categorized in the order *Caudovirales* (tailed phages) and around 60% of these phages with flexible and long tails classified to the family, *Siphoviridae* [55].

### 3.3 Thermal and pH stability of phiPT1

As shown in Table 3, phage phiPT1 was stable from 4 °C to 60 °C upon thermal exposure and not viable at 90 °C. The mean titer of phiPT1 was found to be 8.5 log_10_ PFU/ml upon 1 h treatment at 4 °C, 37 °C, 40 °C, or 60 °C and no significant differences (*p* > 0.05) were recorded among them. Nevertheless, after 1 h incubation at 70 and 80 °C, the rate of survival reduced to 2.5 log_10_ PFU/ml (*p* < 0.05) (Table 3). Moreover, phiPT1 retained a high titer (8.1 log_10_ PFU/ml) from pH 4 to pH 10 for 1 h. However, it exhibited a significant decline (*p* < 0.05) in titer at pH 11 and 12, with phage titers of only 5.5 and 2.4 log_10_ PFU/ml, respectively. No viable virions were at pH 13 suggesting that phiPT1 did not resist strong alkaline condition (*p* < 0.05) (Table 3).

**Table 3:** Thermal and pH stability of phiPT1.

Temperature (C)	Phage titer in log_10_ PFU/mL	*P* value	pH	Phage titer in log_10_ PFU/mL	*P* value
**4**	8.5	*p* > 0.05	2	0	*p* < 0.05
**37**	8.5		3	5.4	
**40**	8.5		4	8.1	*p* > 0.05
**60**	8.5		7	8.1	
**70**	2.5	*p* < 0.05	10	8.1	
**80**	2.5		11	5.5	*p* < 0.05
**90**	0		12	2.4	
**100**	0		13	0	

### 3.4 Multiplicity of infection (MOI)

**Fig. 2:**
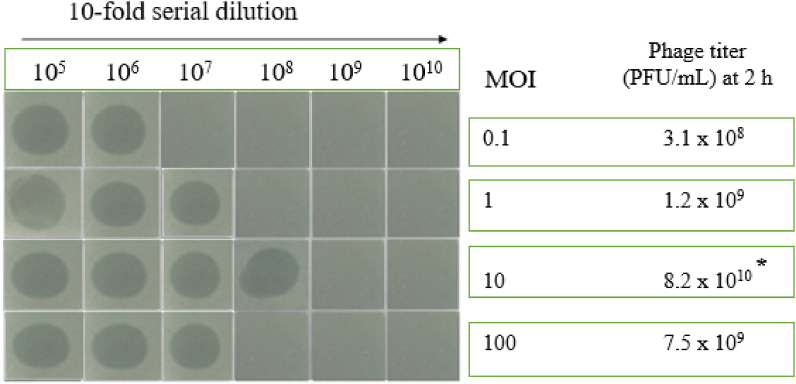
MOI of phiPT1. Asterisk (*) indicates the optimal point of phage infection (i.e., 10 MOI).

The exponential phase culture of *S.* Enteritidis strain PT1 was infected with phiPT1 at different phage titer to determine the MOI. The titer of phiPT1 was measured at 2 h post infection. The results indicated that the optimal MOIs of phiPT1 was found to be 10 which gave the highest production of progeny virion (8.2 × 10^10^ PFU/ml) (Fig. 2).

### 3.5 Phage adsorption and one step growth

**Fig. 3:**
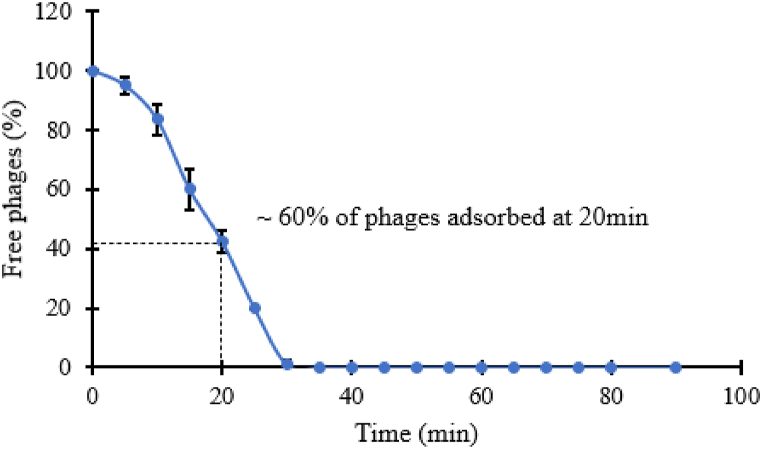
Phage phiPT1 adsorption curve. Each value represents Means ± SD.

We have determined that phage phiPT1 absorbs rapidly on *S*. Enteritidis strain PT1 cells, with nearly 60% of phages were absorbed at 20 min and 100% at 30 min following the infection of host culture with the phiPT1 phage lysate (Fig. 3).

**Fig. 4:**
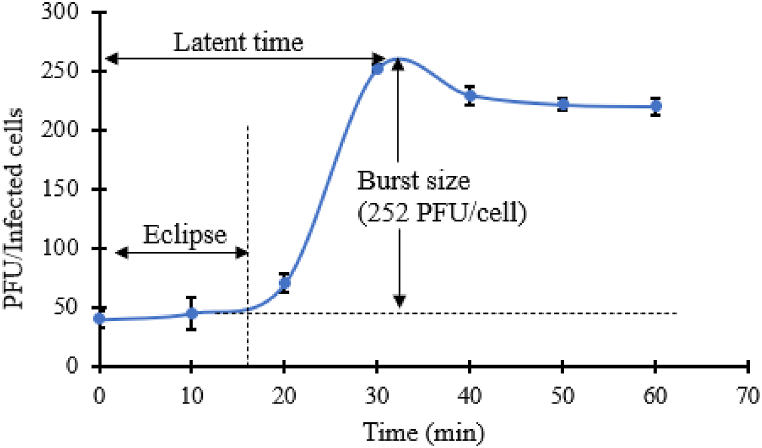
One step growth cycle of phiPT1. Each value represents Means ± SD.

One round phage infection cycle was conducted to assess the latent time and burst size of phiPT1. As shown in Fig. 4, the latent time and burst size of phiPT1 was estimated to be 30 min and 252 PFU/infected cell, respectively (Fig. 4). This result is slightly higher than the previously isolated phage, vB_SalP_TR2, which had 15 min and 211 PFU/cell of latent time and burst size, respectively [56]. In the contrary, the latent period of phiPT1 is shorter than other reported *Salmonella* phages [[57], [58], [59]]. Latent period and burst size are very crucial parameters in the evaluation of the fitness of phages and identification of the potential candidate phages for biocontrol and therapeutic uses [20].

### 3.6 Bacterial reduction assay

**Fig. 5:**
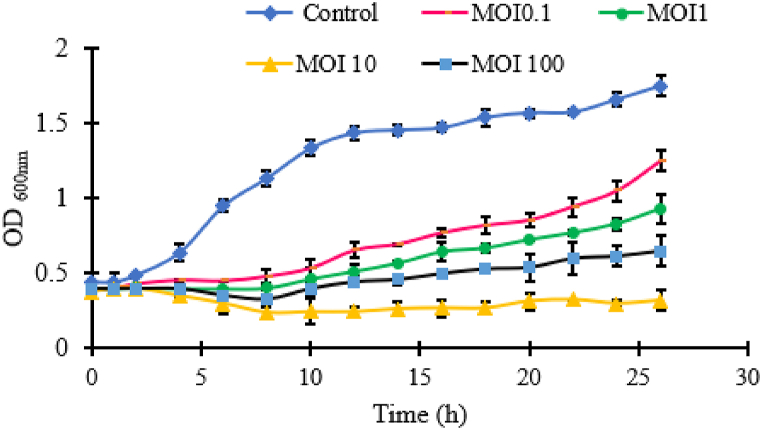
Bacterial killing assay. Each value represents Means ± SD.

The virulence of phage phiPT1 was evaluated against *S.* Enteritidis strain PT1 at different MOIs. phiPT1 phage reduced *Salmonella* proliferation relative to the negative control (MOI 0) at all MOIs (*P* < 0.05). phiPT1 continued to suppress *S.* Enteritidis strain PT1 proliferation up to 24 h post-inoculation except 100 MOI (Fig. 5). At all MOI, re-growth of bacteria was noticed at different time points. Except MOI10 there were no significant differences in the inhibition of the growth of *S.* Enteritidis strain PT1 among the four MOIs (*p* > 0.05). The instability of prolonged bacterial suppression generated by phiPT1 suggests evasion of bacterial phage resistance, a major obstacle in the biocontrol use of these phage [60].

### 3.7 Host range

To determine the killing range of phiPT1, we performed a spot assay and DAL for confirmation. Results have shown that phiPT1 infected 2 of the 11 bacterial species tested (18.2%) (Table 4) suggesting that phiPT1 had a narrow host range. This results is in agreement with the previously reported *Salmonella* phage (vB_SalP_TR2) [56]. The main limitation in utilizing phages as biological control agents for different *Salmonella serovars* is the narrow spectrum of activity of most phages. Most were isolated by targeting *S. enteritidis* and/or *S.* Typhimurium. To overcome these limitations, genetic engineering could be employed to generate a modified phage by upregulating the expression of the desired genes. However, advanced technology is needed [61,62].

**Table 4:** Host range of phage phiPT1.

Bacterial species	Source (Acc.no)	Antimicrobial profile	Spot assay	DAL
*S.* Typhimurium**	KFMRC	MDR	**-**	**-**
*S.* Enteritidis**	(CP016754.1)	MDR	**+**	**+**
*S.* Dublin**	(FJ997268.1)	MDR	**-**	**-**
*S.* Typhi**	(GU826683.1)	MDR	**-**	**-**
*S.* arizonae**	(CP000880.1)	NMDR	**+**	**+**
*S.* Waycross**	(CP022138.1)	MDR	**-**	**-**
S. flexneri*	KFMRC	NMDR	**-**	**-**
E. coli*	KFMRC	MDR	–	**-**
***E. coli ATCC11775***	ATCC	NMDR	–	**-**
***Pseudomonas aeruginosa ATCC9027***	ATCC	NMDR	–	
***Staphylococcus aureus* ATCC12600**	ATCC	MDR	–	**-**
Total lytic positive	**2 (18.2%)**			

“+” = Clear zone/plaques (positive result), “-” = No clear zone/plaques (negative result), Acc.no = Accession number, * Clinical isolate, **food isolate, MDR = Multidrug resistant strain, ATCC = American Type Culture Collection, KFMRC = King Abdulaziz University.

### 3.8 The antimicrobial effect of phage–antibiotics combination

The MIC of *S.* Enteritidis strain PT1 determined to seven antibiotics. The results indicated that the strain was resistant to gentamicin, cefixime, cefepime, ciprofloxacin, and aztreonam, while it was sensitive for colistin and meropenem (Table 5). Before the phage—antibiotic analysis, we assessed the effect of the selected antibiotics on the viability (titer) of the phage phiPT1. Here, we did not find any change on the viability (titer) of phage and no differences in their plaque size and morphology were encountered. We also evaluated the presence of any negative impact on antibiotic associated with phage and no adverse effects were observed. In contrast to our findings, previous report indicated that the treatment of *Escherichia coli* with ΦMFP and T4 phages in combination with cefotaxime affected both the titer of phages and plaque size [30,63].

**Table 5:** MIC and concentration of antibiotics for phage—antibiotic combinations.

Antibiotics	Antibiotic MIC	Selected concentration for PAS study (μg/mL)		
		0.25	0.1	0.01
Gentamicin (≥16)	16	4	1.6	0.16
Cefixime (≥8)	16	4	1.6	0.16
Cefepime (≥4)	4	1	0.4	0.04
Ciprofloxacin (≥1)	1	0.25	0.1	0.01
Aztreonam (≥16)	32	8	3.2	0.32
Meropenem (≥4)	2^a^	0.5	0.2	0.02
Colistin (≥4)	2^a^	0.5	0.2	0.02

a = Sensitive; number without a star indicates resistance.

**Fig. 6:**
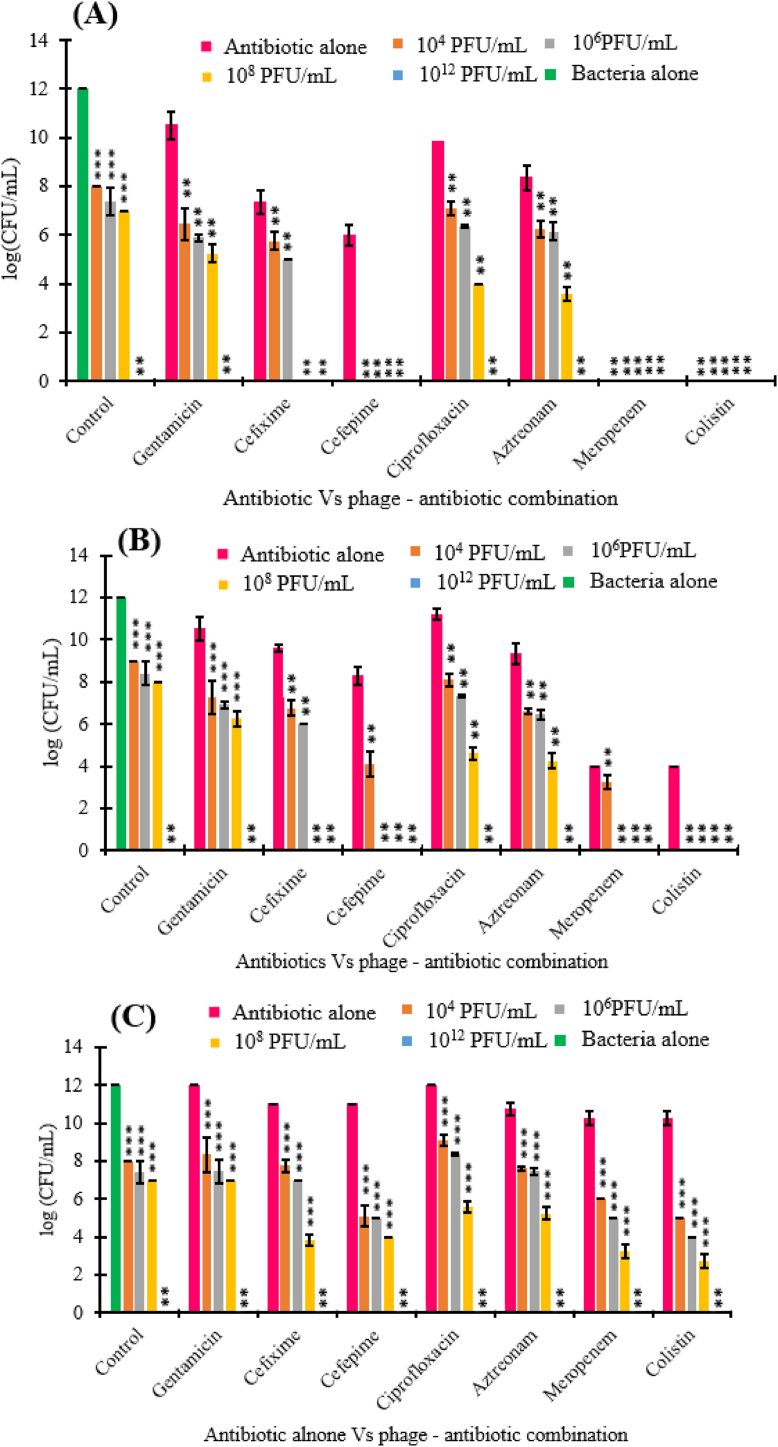
PAS assay results of phage phiPT1 and seven antibiotics at subinhibitory concentration of (A) 0.25 μg/mL; (B) 0.1 μg/mL; (C) 0.01 μg/mL (***p < 0.0001, **p < 0.05). In the control group the green color indicates bacteria culture, the other bar in this group indicates phiPT1 at different concentrations without antibiotics. Viable bacterial densities (X ± SE, three replicates) in 17 h.

The sublethal concentrations were defined based on the results attained from MIC (Table 5). In the PAS assay, we evaluated three different antibiotic concentrations, i.e., 0.25, 0.1 and 0.01 of the MIC. The antibacterial activity of phiPT1 and sublethal antibiotics concentration on *S.* Enteritidis strain PT1 were investigated by computing the viable concentration of cell (CFU/mL) at 17 h of exposure to antibiotic-phage combinations. In the control group (without phage), the concentration of bacterial cell was determined to be 1.2 × 10^12^ CFU/mL at 17 h incubation, while phiPT1 with a final titer of 10^4^, 10^6^ and 10^8^ PFU/mL lessened the bacterial concentration to 5.8, 4.3 and 4.2 log (CFU/mL), respectively (Fig. 6).

In this study, viable host cells were not detected at higher titer of phiPT1 (10^12^ PFU/mL). At sublethal concentrations (0.25 × MIC (0.5 μg/mL)), in the absence of phiPT1, meropenem and colistin resulted in a complete eradication of the bacterial cells. Similarly, without phage, gentamicin (0.25 × MIC (4 μg/mL)) resulted in only a reduction to 10.5 log CFU/mL (*p* < 0.0001). At the same sublethal concentration cefixime (0.25 × MIC (4 μg/mL)), ciprofloxacin (0.25 × MIC (0.25 μg/mL)) and aztreonam (0.25 × MIC (8 μg/mL)) diminished the count of cell to 7.5, 10.2, and 8.4log CFU/mL, respectively (*p* < 0.05). While ceftriaxone lessened the concentration of viable cells to 6log CFU/mL. At 0.1 MIC in the absence of phage, the least reduction of bacterial cells was obtained by ciprofloxacin (11.2 log (CFU/mL)), while meropenem and colistin reduced the cell count to 4log (CFU/mL). At lower concentration of antibiotics (0.01 × MIC), no major effect on the viability of the tested bacterial strain was observed. All in all, out of the seven antibiotics, meropenem and colistin displayed the strongest antimicrobial effect at high sublethal concentration (0.25 × MIC) (Fig. 6).

In the presence of phage, the combination of gentamicin (4 μg/mL) at 0.25 × MIC with the phiPT1 at 10^8^ PFU/mL resulted in a five-log reduction (5.3 log CFU/mL) compared to antibiotics alone (10.3 log CFU/mL) and a two-log diminution in comparison to the phage alone. In the case of ciprofloxacin (0.25 μg/mL, 0.25 × MIC) and aztreonam (8 μg/mL, 0.25 × MIC) - phage treatment (10^8^ PFU/mL phage) resulted in a six and four log reduction of bacterial cells compared to the antibiotics alone, respectively. Interestingly, the other antibiotics cefixime (4 μg/mL), meropenem (0.5 μg/mL), colistin (0.5 μg/mL), and cefepime (1 μg/mL) when combined with phiPT1 (10^8^ PFU/mL) caused a complete clearance of bacterial cells. In addition, the combination of phiPT1 at 10^4^ PFU/mL with ceftriaxone, meropenem and colistin resulted in complete eradication of bacterial cell (Fig. 6A).

PAS assay was next evaluated at 0.1 MIC of the selected antibiotics. At 10^8^ PFU/mL phage titer, cefixime (4 μg/mL), meropenem (0.5 μg/mL), colistin (0.5 μg/mL), and cefepime (1 μg/mL) caused in a full destruction of the host cells. phiPT1 at 10^4^ PFU/mL in combination with ciprofloxacin, gentamicin, cefepime, and aztreonam decreased cell counts approximately by three logs (p < 0.05) (Fig. 6B).

Lastly, the PAS assay was conducted at 0.01 sublethal concentration of the selected antibiotics (Fig. 6C). At this concentration, a dual combination of phiPT1 (10^8^ PFU/mL) and meropenem (0.02 μg/mL, 0.01 × MIC), colistin (0.02 μg/mL, 0.01 × MIC), cefixime (0.16 μg/mL, 0.01 × MIC) and cefepime (0.04 μg/mL, 0.01 × MIC) showed a significant reduction of viable bacterial cells at 17 h incubation (*p* < 0.05) (Fig. 6). When comparing all the tested antibiotics meropenem (MIC of 2) and colistin (MIC of 2), both are inhibitors of the cell wall synthesis which showed good antibacterial activity against the tested organism even at low phage titer. Although *S.* Enteritidis strain PT1 resistant for the tested cephalosporines, it showed synergistic effect with phiPT1. This result may be because of the killing effect of the two agents associated on their action on cell wall of the bacterial cell [45].

The mechanism of action of antibiotics is vary one from the other and even quite different from the action of phages [64]. Thus, the combined effect of phage with antibiotics might vary depending on the antimicrobial potential as well as the nature of these two agents. A study on the combined effect of rifampin, daptomycin, fosfomycin, or ciprofloxacin with *Staphylococcus aureus* phage, Sb-1, studied by Ref. [29] and the results revealed that except one (Fosfomycin) the other antibiotics showed promising inhibitory effect [29]. In a different study, the combined effect of conventional antibiotics and T4 phages resulted in an increased burst size and reduced latent period of the tested phage.

Furthermore, in comparison to cefotaxime alone, the combination of cefotaxime and T4 phage considerably improved the eradication of bacterial biofilm [30]. In accordance with our findings a study reported by Chaudhry et al. indicated that a synergistic effect of phage PA14 in combination with ciprofloxacin (1 × MIC) targeting *P. aeruginosa* was reported [65].

**Fig. 7:**
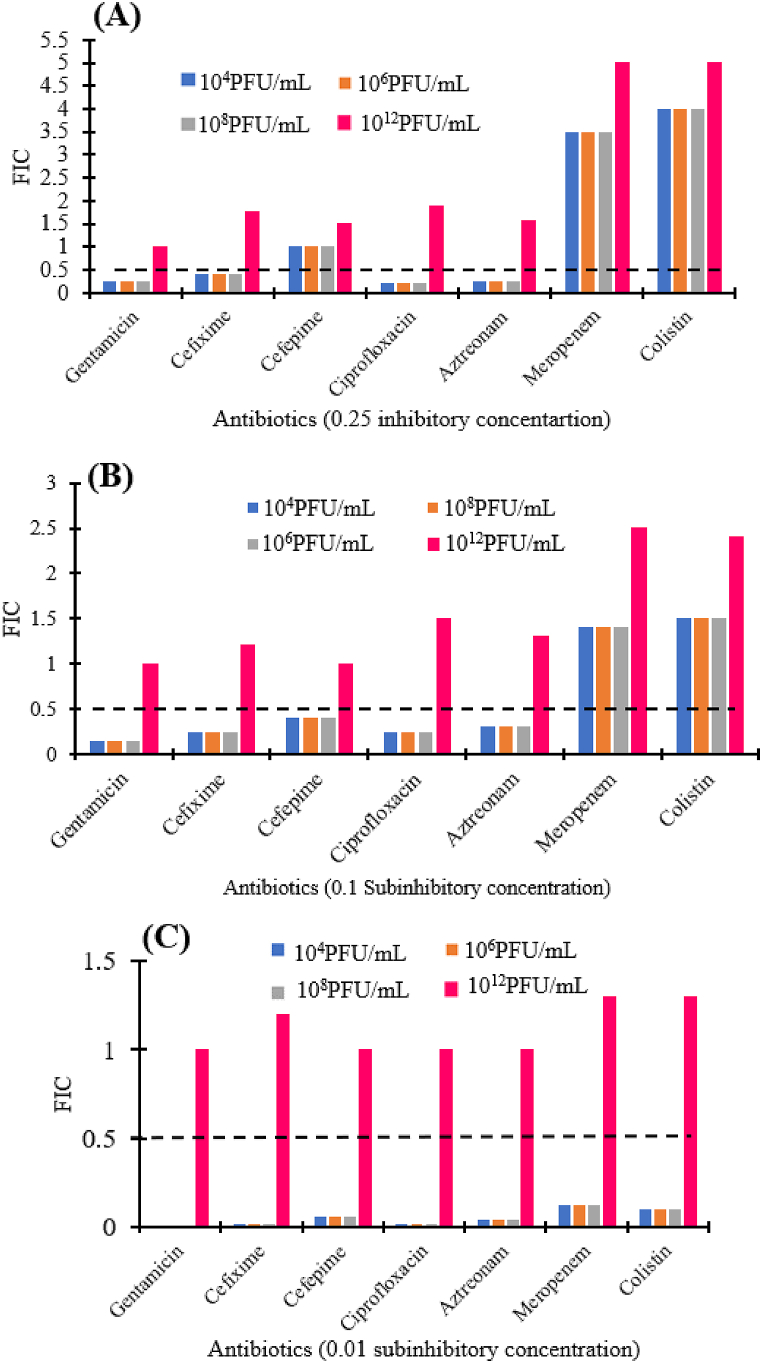
Fractional inhibitory concentration (FIC) of phage–antibiotic combinations. Synergy was determined at three different sublethal concentrations: (A) (0.25 μg/mL), (B) (0.1 μg/mL) and (C) (0.01 μg/mL). FIC was interpreted as antagonistic (ΣFIC >2.0), additive (≥1.0 ΣFIC ≤2.0), indifferent (>0.5 ΣFIC ≤1.0), or synergistic (ΣFIC ≤0.5). Dotted line denotes synergistic effect.

In PAS assay, the synergistic effects were determined by computing the FIC value (ΣFIC ≤0.5). Synergism was not obtained at higher phiPT1 titer (10^12^ PFU/mL) with all tested antibiotics at all subinhibitory concentrations. However, synergistic activities were recorded at 0.25 × MIC of four tested antibiotics: cefixime, gentamicin, ciprofloxacin, and aztreonam in combination with phage at 10^4^, 10^6^ and 10^8^ PFU/mL (ΣFIC ≤0.5) (Fig. 7A). Synergism was detected for all antibiotics (0.1 × MIC) except meropenem and colistin in combination with phiPT1 at 10^4^, 10^6^ and 10^8^ PFU/mL (ΣFIC ≤0.5) (Fig. 7B). Synergism also displayed at the lowest concentrations of all antibiotics (0.01 MIC) in combination with phage at all titers except 10^12^ PFU/mL (Fig. 7C).

As indicated in Fig. 7, four antibiotics namely cefixime, ciprofloxacin, gentamicin, and aztreonam displayed synergistic interaction with phiPT1 (FIC <0.5) at 0.25 of the MIC in all phage titers except 10^12^ PFU/mL. At this subinhibitory concentration, gentamicin (4 μg/mL) combined with phiPT1 at 10^4^, 10^6^, and 10^8^ PFU/mL resulted in a 4, 4.6, and 5.3 log reduction compared to antibiotic alone, respectively. Similarly, at the same subinhibitory concentrations, cefixime (4 μg/mL) resulted in a 1.6, 2.8, and 7.5 log reductions compared to the antibiotics alone. Likewise, ciprofloxacin (0.25 μg/mL) resulted in a 3, 3.8, and 6 log reductions compared to the antibiotics alone (Fig. 6).

PAS also observed at 0.1 of the MIC except for meropenem and colistin in combination with phiPT1 (FIC <0.5) (Fig. 7). A 3.3, 3.6 and 4.3 log reduction were obtained when phiPT1 combined with gentamicin (1.6 μg/mL) at 10^4^, 10^6^, and 10^8^ PFU/mL compared to antibiotic alone, respectively. Similarly, at the same subinhibitory concentration, the combination of phiPT1 and ciprofloxacin (0.1 μg/mL) resulted in a 3, 3.8, and 6.6 log reductions compared to the antibiotics alone. Aztreonam (3.2 μg/mL) and phiPT1 caused a 2.8, 2.9, and 5 log decreases compared to the antibiotic alone (Fig. 6).

When comparing the synergistic effects of the six antibiotics to each other, ciprofloxacin showed the least degree of synergy followed by gentamicin, aztreonam, and cefixime (Figs. 6 and 7). The reason for this may be due to the isolate is highly resistant to these antibiotics or they may have different mechanism of actions in comparison to the action of phiPT1 [45].

In this study, the individual phage therapy showed less lytic activity than the phage-antibiotic combinations especially at the late infection period (Fig. 5). This may be due to the development of phage resistant strains in the third or fourth round of infections. In this regard, phage - antibiotic approach has been effectively reduced the emergence of antibiotic resistant and phage-resistant strains [66]. One of the common examples indicated that the phage mediated degeneration of cell surface receptors responsible for efflux of drugs which result in re-sensitivity of the agent to antibiotic [67]. Numerous *in vitro* investigations validate the re-sensitivity to antimicrobial agents when they were mixed with phages [68,69]. In addition, PAS alleviate the required concentration of antibiotics in comparison to the treatment using mono-antibiotic [66].

Even though the detailed mechanism of PAS is yet to be elucidated, the activity of phages could be enhanced by antibiotics in three main different mechanisms which are mainly induced by the change in the morphology of the bacterium (elongation/filamentation). These mechanisms are acceleration of cell lysis (increase sensitivity to lytic enzymes, increase the expression of lytic enzymes), increase the burst size of phages, and increase phage adsorption rate by upregulating the expression of receptors [70,71].

## 4 Conclusions

In this study, one lytic phage, phiPT1, isolated against extended-spectrum beta-lactam producing *Salmonella* Enteritidis derived from chicken carcass belonging to the family *Siphoviridae*. phiPT1 had a narrow host range, small latent time, and high burst size. In addition, it showed wide pH and thermal tolerance, and virulent against the tested host strain. The phage-antibiotic combination analysis indicated that PAS was not detected at higher phiPT1 titer (10^12^ PFU/mL) as it killed the bacteria cells alone without the involvement of antibiotics, while synergism was observed at 0.25, 0.1 and 0.01 subinhibitory concentration of the tested antibiotics mixed at 10^4^, 10^6^ and 10^8^ PFU/mL of phiPT1, respectively. In general, the results obtained in this study can serve as a basis for further investigation in the therapeutic applications of phages alone and in combination with conventional antibiotics to control *S*. Enteritidis infections. In this regard, additional investigations are required to assess the potential of phiPT1 individually and in combination of antibiotics against the planktonic cell and their biofilms *in vivo* experiments. In addition, the mechanism of action and related pharmacological mechanisms of PAS should be studied for better success.

## Author contribution statement

Rashad R. Al-Hindi: Conceived and designed the experiments; Analyzed and interpreted the data; Contributed reagents, materials, analysis tools or data; Wrote the paper.

Mona G. Alharbi: Analyzed and interpreted the data; Contributed reagents, materials, analysis tools or data.

Ibrahim A. Alotibi; Sheren A. Azhari; Reem M. Farsi: Performed the experiments; Analyzed and interpreted the data.

Addisu D. Teklemariam: Conceived and designed the experiments; Performed the experiments; Analyzed and interpreted the data; Wrote the paper.

## Funding statement

Prof. Rashad R. Al-Hindi was supported by Institutional Fund Projects [IFPRC-109-130-2020].

## Data availability statement

Data included in article/supp. material/referenced in article.

## Declaration of interest's statement

The authors declare no competing interests.
